# Daratumumab‐based regimens versus CyBorD in newly diagnosed patients with AL amyloidosis and IIIb cardiac stage: A matched case‐control study

**DOI:** 10.1002/hem3.70112

**Published:** 2025-03-24

**Authors:** Claudia Bellofiore, Marco Basset, Giuseppe Damiano Sanna, Andrea Foli, Roberta Mussinelli, Martina Nanci, Alessandro Fogliani, Martina Ciardo, Mario Nuvolone, Giampaolo Merlini, Giovanni Palladini, Paolo Milani

**Affiliations:** ^1^ Department of Molecular Medicine University of Pavia Pavia Italy; ^2^ Amyloidosis Research and Treatment Center, Fondazione IRCCS Policlinico San Matteo Pavia Italy; ^3^ Clinical and Interventional Cardiology Sassari University Hospital Sassari Italy

Immunoglobulin light chain (AL) amyloidosis is a life‐threatening systemic disease especially when the heart is severely affected.^1^ The pathogenic mechanism relies on the production, by a B‐cell clone, of immunoglobulin free light chains (FLC), which form fibrillar structures that deposit in organs and tissues and exert cardiac toxicity.^2^ The heart is the most frequently affected organ and is the major determinant of clinical outcome.^1^ The current prognostic cardiac staging system stratifies patients' survival using two biomarkers: troponins and B‐type natriuretic peptide (BNP) or N‐terminal proBNP (NT‐proBNP).^3^, ^4^ Approximately 20% of patients present with advanced heart involvement, classified as IIIb cardiac stage. These patients have a dismal survival^3^, ^5^ that has not improved in recent years.^1^ The standard frontline treatment for AL amyloidosis has been bortezomib in combination with dexamethasone and alkylating agents (i.e., cyclophosphamide [CyBorD]^5^ or melphalan)^6^ until the approval of daratumumab in combination with CyBorD (Dara‐CyBorD) in 2021, which significantly improved the rate and depth of hematologic response, as well as overall survival (OS), as reported by the ANDROMEDA trial.^7^, ^8^ However, stage IIIb patients were excluded from the pivotal study and the best upfront treatment strategy for these patients remains to be clarified. For this reason, daratumumab was not licensed for use in stage IIIb patients in Europe. Nevertheless, the European Hematology Association and International Society of Amyloidosis (EHA‐ISA) working group guidelines recommend, where feasible, the use of daratumumab, even as monotherapy, in stage IIIb patients based on the encouraging preliminary data of the EMN22 phase II multicenter study (NCT04131309).^9^, ^10^ Several retrospective series have evaluated daratumumab‐containing regimens in treatment‐naïve stage IIIb patients reporting promising results.^11^, ^12^ However, all available data on daratumumab in this high‐risk population derive from uncontrolled studies, and head‐to‐head comparative data remain limited. The only comparative study to date, conducted by Oubari et al.,^13^ matched patients solely by disease stage, underscoring the need for further comparative studies to evaluate the effectiveness of daratumumab in this setting.

We designed the present retrospective case‐control study to assess the efficacy of daratumumab‐based therapies versus CyBorD as an upfront treatment for newly diagnosed AL amyloidosis with IIIb cardiac stage. The prospectively maintained databases of the Amyloidosis Research and Treatment Center of Pavia were searched for newly diagnosed patients with systemic AL amyloidosis and IIIb cardiac stage evaluated between January 2012 and December 2022. Data were collected from the Ethics Committee‐approved ReAL amyloidosis registry (NCT04839003), and all patients gave written informed consent. Patients and controls were matched, at a 1:1 ratio, for the following variables: age, cardiac biomarkers (NT‐proBNP and high‐sensitivity troponin I), estimated glomerular filtration rate, 24‐h proteinuria, sFLC, and bone marrow plasma cell (BMPC) infiltrate. In Italy, Dara‐CyBorD was approved as a frontline treatment for AL amyloidosis in January 2023 and until that date, daratumumab combinations were accessible solely to patients with AL amyloidosis and a BMPC infiltrate of at least 10%. Thus, all patients included in this study have a BMPC infiltrate ≥ 10%. Patients with myeloma‐defining events (MDEs), defined according to the International Myeloma Working Group criteria, were excluded. Hematologic and organ responses were assessed according to the International Society of Amyloidosis criteria 3 and 6 months after treatment initiation.^14^ Cardiac responses were further graded according to criteria proposed by Muchtar et al.^15^ The analysis of response was by an intent‐to‐treat approach: patients who died before response evaluation were considered non‐responders. Differences in hematologic and cardiac response rates between patients and controls were tested for significance using Fisher's exact test. Major organ deterioration event‐free survival (MOD‐PFS) was defined as the time from treatment initiation to any one of the following events, whichever comes first: death, starting of a second line of therapy, end‐stage cardiac or renal disease, or organ or hematologic progression as per consensus guidelines.^14^ Survival curves were plotted according to Kaplan–Meier, and differences in overall OS and in MOD‐PFS were tested for significance using the log‐rank test. Statistical analysis was conducted with MedCalc® Statistical Software version 20.027 (MedCalc Software Ltd; https://www.medcalc.org; 2022).

The whole study population comprised 62 matched patients. The daratumumab cohort is composed of all 31 consecutive stage IIIb subjects treated with daratumumab‐based regimens between 2021 and 2022. Thirteen patients (41%) received daratumumab in combination with bortezomib (11 [35%] associated with melphalan and dexamethasone; 2 [6%] Dara‐CyBorD through compassionate use), 11 (35%) with lenalidomide, and 7 (22%) were treated with daratumumab monotherapy. They were matched with 31 controls searched from a total of 71 subjects treated with CyBorD diagnosed between 2012 and 2022. Although this time gap could introduce variability in supportive therapies, cardiac support strategies did not differ significantly between the two cohorts (Supplementary Table 1). In all cases, dexamethasone was started at 20 mg weekly, and bortezomib was started at 0.7 mg/m^2^ weekly and escalated up to 1.3 mg/m^2^ according to tolerability.

Baseline patients' characteristics are listed in Table 1. The median follow‐up of living patients was 28 months (95% confidence interval [CI] 14–38 months). Median OS of the entire cohort was 4.6 months (95% CI 3–55 months), and patients of the daratumumab cohort had a significantly prolonged OS (10.3 vs. 4.0 months, hazard ratio [HR] 2.13, 95% CI 1.19–3.81, *p* = 0.010, Figure 1). Median MOD‐PFS was significantly longer with daratumumab combinations compared to CyBorD (10.2 vs. 3.2 months; HR 2.7; 95% CI 1.50–4.96, *p* < 0.001, Figure 1). No significant differences in early mortality rates, within 1 and 3 months from diagnosis, were observed between the daratumumab and the control cohort (3% vs. 9%; *p* = 0.362; 32% vs. 35%, *p* = 0.796).

**Table 1:** Patients' characteristics.

	Dara‐based (*N* = 31) median (IQR), *n* (%)	CyBorD (*N* = 31) median (IQR), *n* (%)	*p*
Median age, yr	69 (63–73)	64 (59–69)	0.074
Male sex	21 (67)	22 (70)	0.791
Organ involvement			
Kidney	16 (51)	11 (35)	0.214
Liver	0 (0)	3 (9)	0.118
Renal stage			
I/II/III	4 (25)/7 (43)/3 (18)	0 (0)/8 (72)/3 (27)	0.132
Dialysis at diagnosis	2 (12)	0 (0)	0.245
Biomarkers			
Abnormal ALP	4 (13)	4 (13)	0.999
ALP, UI/L	86 (73–108)	105 (95–139)	0.095
NT‐proBNP, ng/L	12,660 (9953–15,965)	14,800 (10,882–26,081)	0.149
NT‐proBNP, mean ± SD	16,432 ± 14,192	20,346 ± 14,230	0.282
hs‐TnI, ng/L	240 (150–390)	288 (164−451)	0.490
eGFR > 60 mL/min/1.73 m^2^	15 (48)	15 (48)	0.999
eGFR, mL/min/1.73 m^2^	65 (44 to >60)	45 (36 to >60)	0.103
Proteinuria, g/24 h	0.69 (0.29–3.50)	0.44 (0.18–3.40)	0.482
Involved light‐chain type			
*κ*:*λ*	5 (16):26 (83)	7 (22):24 (77)	0.542
dFLC, mg/L	384 (184–1126)	261 (184–673)	0.326
dFLC > 180 mg/L	23 (74)	23 (74)	0.999
BMPC, %	18 (13–24)	15 (11–22)	0.247
BMPC > 10%	31 (100)	31 (100)	0.999
SBP, mmHg	106 (105–135)	109 (101–123)	0.406
DBP, mmHg	74 (69–80)	71 (64–76)	0.394
IVS thickness, mm	16 (15–17.5)	15 (11−17)	0.028
LVEF, %	45 (43–57)	52 (48–59)	0.084

Abbreviations: ALP, alkaline phosphatase; BMPC, bone marrow plasma cells; DBP, diastolic blood pressure; dFLC, difference between involved and uninvolved serum‐free light chains; eGFR, estimated glomerular filtration rate; hs‐TnI, high‐sensitivity cardiac troponin I; ISV, interventricular septum; LVEF, left ventricular ejection fraction; NT‐proBNP, N‐terminal pro‐brain natriuretic peptide; SBP, systolic blood pressure; SD, standard deviation.

**Figure 1:**
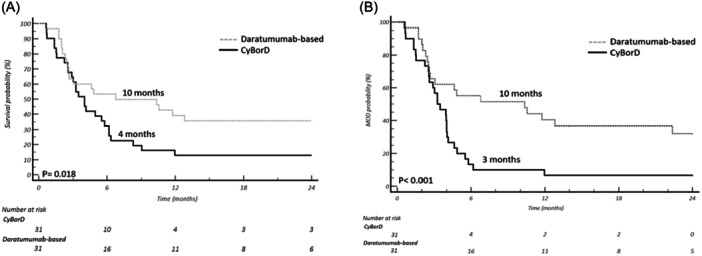
**(A) Median overall and (B) major organ deterioration progression‐free survival (MOD‐PFS) in the two cohorts of patients**.

At 3 months from treatment initiation, the overall hematologic response rate of the daratumumab cohort was 50% (complete response [CR] 12%, very good partial response [VGPR] 29%, partial response [PR] 9%) compared to 21% (CR 6%, VGPR 9%, PR 6%) of the control group, *p* = 0.034. A higher proportion of profound hematologic response (≥VGPR) was observed in the daratumumab cohort (41% vs. 16%; *p* = 0.048). No statistically significant differences in deep hematologic response rates (≥VGPR) were observed among patients treated with daratumumab in combination with bortezomib (46%), lenalidomide (27%), or as monotherapy (57%), *p* = 0.205. Cardiac response, at 3 months from treatment initiation, was significantly more frequent and deeper in patients treated with daratumumab (eight [25%], three cardiac VGPR, and five cardiac PR) compared to controls (one [3%] cardiac PR), *p* = 0.026. At 6 months, similar response patterns were observed: the hematologic overall response rate was 48% in the daratumumab‐treated group compared to 19% in the CyBorD group (≥VGPR: 42% vs. 18%; *p* = 0.030), and cardiac responses occurred in 29% of daratumumab‐treated patients (*n* = 9; three cardiac VGPR, six cardiac PR) compared to 3% in the control group (*n* = 1; cardiac PR), *p* = 0.005.

In our series, the rate of profound hematologic response at 3 months in patients receiving daratumumab was comparable to the one reported by the EMN22 phase II study (≥VGPR 48%) and Theodorakakou et al. (≥VGPR 50%).^9^, ^12^ However, this finding differs from two recently published studies in which the rate of deep hematologic response (≥VGPR) was higher.^11^, ^13^ In particular, in a case series of 19 patients described by Chakraborty et al., more than 90% of IIIb cardiac stage patients treated with dose‐modified Dara‐CyBorD achieved a VGPR or better at 3 months from treatment initiation.^11^ Nevertheless, we cannot directly compare the rate of hematologic response to the other series because 22% of our patients received daratumumab in combination with lenalidomide. The heterogeneity of daratumumab regimens used in our study reflects common clinical practice in the treatment of patients with AL amyloidosis, especially before Dara‐CyBorD approval.^12^, ^13^, ^16^

The present work is the first matched case‐control study evaluating the efficacy of daratumumab‐based regimens versus CyBorD in newly diagnosed patients with IIIb cardiac stage and high plasma cell burden. Despite all patients included in our study having a BMPC ≥ 10%, none exhibited MDEs. We found that daratumumab‐containing therapies were associated with prolonged OS compared to CyBorD in this frail population. This finding aligns with recent data from the ANDROMEDA study in the non‐stage IIIb population and corroborates the report by Oubari et al., which demonstrated significantly improved OS with daratumumab‐based regimens compared to bortezomib‐based regimens.^8^, ^13^ Notably, the median OS of 10 months observed in our cohort closely parallels the outcomes reported in the phase 2 EMN22 clinical trial.^9^ However, as in the latter study, daratumumab‐based regimens in our series did not lead to an improvement in early mortality rates. The 1‐ and 3‐month mortality rates were consistent with those reported in the EMN22 trial (9% and 27.5%, respectively),^9^ while the 1‐month mortality rate was lower than the 25% reported in the study by Shen et al.^17^

In conclusion, the results of this matched case‐control analysis demonstrate that even in stage IIIb patients with a relatively high clonal burden, the addition of daratumumab in the frontline setting results in higher rates of deeper hematologic and cardiac response, which are associated with prolonged OS. Taken together, these observations emphasize the critical need to broaden access to daratumumab for stage IIIb patients. While randomized prospective trials are lacking, the growing body of evidence strongly supports the integration of daratumumab into clinical practice for this high‐risk population.

## AUTHOR CONTRIBUTIONS

Claudia Bellofiore wrote the manuscript, collected data, and analyzed and interpreted data. Marco Basset, Giuseppe Damiano Sanna, Andrea Foli, Roberta Mussinelli, Martina Nanci, Alessandro Fogliani, Martina Ciardo, and Mario Nuvolone evaluated patients. Giovanni Palladini and Paolo Milani designed the study, wrote the manuscript, and evaluated patients. Giampaolo Merlini evaluated patients and critically reviewed the manuscript and gave the final approval.

## CONFLICT OF INTEREST STATEMENT

P. M.: Pfizer (honoraria for lectures), Janssen‐Cilag (honoraria for lectures and advisory board), Siemens (advisory board). M. B.: Janssen (honoraria for lectures). G. D. S.: Cardiac ATTR Amyloidosis Fellowship grant funded by the International Society of Amyloidosis. Ma. Nu.: Janssen (honoraria for lectures). An. F.: Janssen (honoraria for lectures). G. P.: advisory boards for Alexion, Argobio, Janssen, and Protego; honoraria from Alexion, Argobio, Janssen, Protego, The Binding Site, Pfizer, Prothena, Sebia, and Siemens; research funding from Gate Bioscience and The Binding Site. All remaining authors have declared no conflict of interest.

## ETHICS STATEMENT

Patients' data were drawn from the Ethics Committee‐approved ReAL amyloidosis registry (NCT04839003). All patients had given written informed consent before inclusion in ReAL.

## FUNDING

The authors received no financial support for the research, authorship, and/or publication of this article.

## Supporting information

Supporting information.

## Data Availability

The data that support the findings of this study are available from the corresponding author upon reasonable request. For original data, please contact giovanni.palladini@unipv.it.
