# Supplementary material for: Daratumumab‐based regimens versus CyBorD in newly diagnosed patients with AL amyloidosis and IIIb cardiac stage: A matched case‐control study
Source: Hemasphere. 2025 Mar 24;9(3):e70112. doi: 10.1002/hem3.70112 (PMC11931317; doi:10.1002/hem3.70112)
Supplement: Supplementary file 1 — Supporting information. [file HEM3-9-e70112-s001.docx]

**Supplementary Materials**

**Table. 1** Cardiological support therapies across the study cohorts

| **Daratumumab-cohort (N=31)** | | | | **CyBorD cohort (N=31)** | | | | |
| --- | --- | --- | --- | --- | --- | --- | --- | --- |
| **Class of drugs** | **N (%)** | **Drug** | **N (%)** | **Class of drugs** | **N (%)** | **Drug** | **N (%)** | ***p*** |
| Beta-blockers | 13 (42%) | Bisoprolol | 12 (38%) | Beta-blockers | 11 (36%) | Bisoprolol | 8 (25%) | .641 |
|  |  | Nebivol | 5 (16%) |  |  | Nebivol | 0 |  |
|  |  | Carvediolol | 1 (3%) |  |  | Carvediolol | 3 (9%) |  |
| Diuretics and MRAs | 25 (80%) | Furosemide | 24 (77%) | Diuretics and MRAs | 27 (87%) | Furosemide | 27 (87%) | .561 |
|  |  | Spironolactone | 5 (16%) |  |  | Spironolactone | 6 (19%) |  |
|  |  | Potassium canrenoate | 16 (51%) |  |  | Potassium canrenoate | 11 (36%) |  |
|  |  | Furosemide + Spironolactone | 0 |  |  | Furosemide + Spironolactone | 1 (3%) |  |
| ACE-inhibitors | 8 (25%) | Ramipril | 5 (16%) | ACE-inhibitors | 4 (13%) | Ramipril | 2 (6%) | .220 |
|  |  | Enalapril | 2 (6%) |  |  | Enalapril | 2 (6%) |  |
|  |  | Zofenopril | 1 (3%) |  |  | Zofenopril | 0 |  |
| Angiotensin II receptor antagonists | 2 (6%) | Olmesartan | 2 (6%) | Angiotensin II receptor antagonists | 0 | Olmesartan | 0 | .245 |
| Gliflozins | 1 (3%) | Canagliflozin | 1 (3%) | Glifozins | 0 | Canagliflozin | 0 | .500 |

Legend: ACE-inhibitors, Angiotensin-converting enzyme inhibitors; MRAs, mineralocorticoid receptor antagonist.
